# Temperature dependence of ssrA-tag mediated protein degradation

**DOI:** 10.1186/1754-1611-6-10

**Published:** 2012-07-23

**Authors:** Oliver Purcell, Claire S Grierson, Mario di Bernardo, Nigel J Savery

**Affiliations:** 1 MIT Synthetic Biology Center, Massachusetts Institute of Technology, 500 Technology Square, NE47-235, Cambridge, MA 02139 USA; 2 School of Biological Sciences, University of Bristol, Bristol BS8 1UG, UK; 3 Department of Engineering Mathematics, University of Bristol, Bristol BS8 1TR, UK; 4 Department of Systems and Computer Engineering, University of Naples Federico II, Via Claudio 21, Napoli, 80125, Italy; 5 School of Biochemistry, University of Bristol, Bristol BS8 1TD, UK

## Abstract

Building synthetic gene networks with highly transient dynamics requires rapid protein degradation. We show that the degradation conferred by two commonly used ssrA tags is highly temperature dependent. Synthetic gene networks are being used increasingly in real-world applications where they may be subjected to variable conditions, and be required to display precise, quantitative dynamics, which will be more susceptible to environmental changes than the general qualitative dynamics focussed on so far.

## Introduction

Rapid protein degradation is vital for the creation of synthetic gene networks with highly transient dynamics. The standard means of achieving rapid protein degradation in bacterial synthetic gene networks has been through the use of ssrA tags. These are short (usually 11 amino acid) peptide tags added cotranslationally to the C-terminus of a nascent protein, that are recognised by the AAA+ ClpXP and ClpAP host proteases [1] and target the tagged protein for degradation [2,3]. ssrA tags are part of a larger family of degradation tags, or ‘degrons’, which can be present in both the N- and C- terminus of proteins [4]. The ssrA tags are generally referred to by the final three amino acids, for instance the wild-type tag ends in the amino acid sequence LAA and is known as the LAA-ssrA tag. ssrA tags have been used frequently in synthetic oscillators [5-8].

Synthetic biology is increasingly moving towards real-world applications [9] in which synthetic gene networks will not always be present at favourable laboratory conditions. Networks designed under one set of conditions (growth rate, nutrient supply, temperature etc) will not necessarily function as designed under another set of conditions. Networks must therefore be designed to function correctly, or at least predictably, in sub-optimal and changeable environments. A robustness to temperature variation will need to form part of this, as demonstrated by the observation of a substantial effect of temperature on a synthetic gene oscillator [6]. It has recently been shown that the bacterial chemotaxis machinery is highly temperature compensated [10]. Therefore a relevant question is whether the bacterial degradation machinery also exhibits a significant level of temperature compensation. This is important not only for synthetic biologists investigating the effects of temperature on gene network performance, but also for those studying the degradation machinery itself.

Here we examine the degradation of LacI, a protein frequently used in bacterial synthetic gene networks [5,6,8,11-13], tagged with either the LAA or LVA ssrA tag. We present results that demonstrate a marked temperature dependence in the degradation rate conferred by both tags.

## Results and discussion

**Figure 1:**
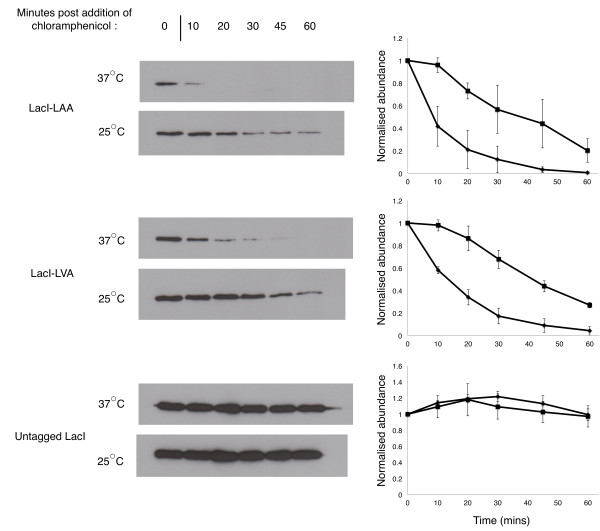
**Degradation of ssrA-tagged and untagged LacI at 25°C and 37°C.** This figure shows representative western blots and quantification of the levels of LacI at 0 minutes (prior to the addition of chloramphenicol) and 10-60 minutes after addition of chloramphenicol in *E. coli* M182 grown at 25°C (squares) and 37°C (diamonds). Sample loading was normalised by culture OD. LacI levels are shown relative to the amount present at *t*=0, are the mean of three independent experiments and are shown with standard error.

Figure 1 shows the amount of LacI, tagged with either an LAA (LacI-LAA) or LVA (LacI-LVA) ssrA tag, or left untagged, from *E. coli* cells grown at 37 or 25°C, before the addition of chloramphenicol (used to stop translational elongation) (0 minutes) and at 10, 20, 30, 45 and 60 minutes post addition of chloramphenicol.

LacI carrying an LAA or LVA tag was degraded approximately 3-5 fold faster in cells grown at 37°C than in cells grown at 25°C. The apparent half-life of LacI-LAA increased from ∼8 minutes at 37°C to ∼38 minutes at 25°C and the apparent half-life of LacI-LVA increased from ∼13 minutes to ∼41 minutes. The stability of untagged LacI was unaffected by temperature, indicating that the observations were dependent on the presence of the ssrA tags.

The result presented here demonstrates that degradation conferred by both LAA and LVA ssrA tags is substantially affected by temperature, and shows that little or no mechanism for temperature compensation exists in this system. Degradation of some tagged cellular proteins has been shown to be temperature dependent [14], but to the best of our knowledge the effect of tempe- rature on the stability of proteins tagged with ssrA tags commonly used within synthetic biology has not been previously investigated. This temperature dependence has been demonstrated for LacI, and there is no reason to assume that it will not extend to other proteins.

This result is potentially important for the future construction of synthetic gene networks in bacteria. Future networks will be increasingly designed for real-world applications where they may need to function over a range of temperatures and conditions. This result highlights a limitation of the current means of tuning protein degradation within bacterial synthetic gene networks and suggests that new, perhaps synthetic, protein degradation pathways that are robust to environmental (and physiological) variations need to be developed.

## Materials and methods

*E. coli* strain M182 [15] was transformed with one of three plasmids containing *lacI* tagged with either an LAA (amino acid sequence: AANDENYALAA. DNA sequence: GCTGCTAACGACGAAAACTACGCTCTGGCTGCT) or LVA (amino acid sequence: AANDENYALVA. DNA sequence: GCTGCAAACGACGAAAACTACGCTTTA- GTAGCT) ssrA tag, or left untagged, referred to as pOP_L-LAA, pOP_L-LVA and pOP_L-NoTg respectively. The plasmids were constructed by cloning *lacI* and its two tagged variants into an expression cassette comprising the *Prrn* promoter and the U0 RBS region [16], upstream of the *lacI* coding region, and the terminator BBa_B0015 [17] downstream. The plasmid backbone was pSB6A1 [18]. Transformed cells were grown overnight on LB agar with ampicillin (100*μ*g/ml). Colonies were grown in 5 ml of EZ rich media (Teknova, CA) overnight. 0.5 ml of culture was added to 25 ml EZ rich media, and grown at both 25 and 37°C with shaking (250 rpm) to OD_600_ of 0.4-0.6. A 1 ml sample was taken, centrifuged at 11,700x g and supernatant removed. The pellet was frozen in liquid nitrogen. Chloramphenicol was added to the cultures to a final concentration of 136 *μ*g/ml. 1 ml samples were taken at subsequent time points of 10, 20, 30, 45 and 60 minutes. The same procedure for the initial time point sample was followed. Samples were stored at -80°C.

To assay LacI abundance, SDS loading buffer was added to samples in an amount relative to the OD of the sample. Samples were heated for 3 minutes at 95°C, before being analysed by SDS-PAGE and western blotting, using a mouse monoclonal antibody against LacI (Abcam) and a goat anti-mouse IgG-HRP conjugated secondary antibody (Santa Cruz Biotechnology). Blots were quantified using ImageJ software.

## Authors’ contributions

OP, NJS, CSG and MdB conceived the experiments. OP carried out the experiments. OP and NJS wrote the manuscript. All authors read and approved the final manuscript.
